# Effects of aging on liver microcirculatory function and sinusoidal phenotype

**DOI:** 10.1111/acel.12829

**Published:** 2018-09-08

**Authors:** Raquel Maeso‐Díaz, Martí Ortega‐Ribera, Anabel Fernández‐Iglesias, Diana Hide, Leticia Muñoz, Amelia J. Hessheimer, Sergi Vila, Rubén Francés, Constantino Fondevila, Agustín Albillos, Carmen Peralta, Jaime Bosch, Frank Tacke, Victoria C. Cogger, Jordi Gracia‐Sancho

**Affiliations:** ^1^ Liver Vascular Biology Research Group, Barcelona Hepatic Hemodynamic Laboratory IDIBAPS Biomedical Research Institute, University of Barcelona Medical School Barcelona Spain; ^2^ Biomedical Research Networking Center in Hepatic and Digestive Diseases (CIBEREHD) Madrid Spain; ^3^ Immune System Diseases Laboratory, Department of Medicine University of Alcalá Alcalá de Henares Spain; ^4^ Liver Surgery and Transplantation Unit IDIBAPS, Hospital Clínic de Barcelona Barcelona Spain; ^5^ Instituto de Investigación Sanitaria y Biomédica de Alicante (ISABIAL – Fundación FISABIO) Alicante Spain; ^6^ Department of Gastroenterology and Hepatology Hospital Universitario Ramón y Cajal, IRYCIS, Universidad de Alcalá Madrid Spain; ^7^ Protective Strategies Against Hepatic Ischemia‐Reperfusion Group IDIBAPS Barcelona Spain; ^8^ Hepatology, Department of Biomedical Research Inselspital, Bern University Bern Switzerland; ^9^ Dept of Medicine III University Hospital Aachen Aachen Germany; ^10^ Centre for Education and Research on Ageing & ANZAC Research Institute University of Sydney and Concord Hospital Sydney Australia

## Abstract

The socioeconomic and medical improvements of the last decades have led to a relevant increase in the median age of worldwide population. Although numerous studies described the impact of aging in different organs and the systemic vasculature, relatively little is known about liver function and hepatic microcirculatory status in the elderly. In this study, we aimed at characterizing the phenotype of the aged liver in a rat model of healthy aging, particularly focusing on the microcirculatory function and the molecular status of each hepatic cell type in the sinusoid. Moreover, major findings of the study were validated in young and aged human livers. Our results demonstrate that healthy aging is associated with hepatic and sinusoidal dysfunction, with elevated hepatic vascular resistance and increased portal pressure. Underlying mechanisms of such hemodynamic disturbances included typical molecular changes in the cells of the hepatic sinusoid and deterioration in hepatocyte function. In a specific manner, liver sinusoidal endothelial cells presented a dysfunctional phenotype with diminished vasodilators synthesis, hepatic macrophages exhibited a proinflammatory state, while hepatic stellate cells spontaneously displayed an activated profile. In an important way, major changes in sinusoidal markers were confirmed in livers from aged humans. In conclusion, our study demonstrates for the first time that aging is accompanied by significant liver sinusoidal deregulation suggesting enhanced sinusoidal vulnerability to chronic or acute injuries.

## 1 INTRODUCTION

Societies in developed countries are getting older due to the increase in life expectancy. Nowadays, 14% of European citizens are aged over 65, and by 2030, they are expected to constitute 23% (He, Goodkind, & Kowal, 2016). Therefore, the pace of population aging represents an important healthcare and social issue and it is therefore essential to understand the molecular basis of aging to identify possible approaches for therapeutic intervention.

The liver plays essential roles in metabolism, toxicants clearance, regulation of inflammation, and molecule biosynthesis. To fulfill these complex tasks, it requires an adequate microcirculation and a correct coordinated function of all hepatic cell types (Arias et al., 2009). Although it is well known that hepatocytes constitute the main cell type contributing to the metabolic and synthetic capacities of the liver, their function deeply depends on an efficient exchange of substances with the blood stream and a proper communication with other hepatic cells.

Liver sinusoidal endothelial cells (LSEC), hepatic stellate cells (HSC), and Kupffer cells (KC) are the major components of the hepatic sinusoid, which collaborate to maintain the integrity and functionality of the unique liver microcirculatory system (Marrone, Shah, & Gracia‐Sancho, 2016). LSEC are a very specialized fenestrated endothelial cell forming the capillary bed of the sinusoids, being separated from hepatocytes through the space of Disse. HSC are vitamin A storing cells located in the space of Disse, surrounding LSEC, and represent the liver pericytes. At last, KC, the resident macrophages of the liver, are located at the luminal side of the endothelial lining (Fernández‐Iglesias & Gracia‐Sancho, 2017; Friedman, 2008; Ju & Tacke, 2016).

Previous studies characterized single variables in the liver during aging and showed reduction in liver mass and hepatic blood flow, partial loss of endothelial fenestration, and possibly activation of HSC (Le Couteur et al., 2001; Vollmar, Pradarutti, Richter, & Menger, 2002). Nevertheless, studies analyzing in depth the hepatic sinusoid, its cellular components, and the hepatic microcirculatory function in aging are limited.

This study aimed at comprehensively characterizing the phenotype of the sinusoid in aged liver using a preclinical rat model of healthy aging, particularly focusing on the microcirculatory function and the molecular profile of each major liver cell type. In addition, our study further compared these findings to the key cellular modifications related to aging in livers from young and aged humans.

## 2 RESULTS

### 2.1 Aged rat model: Baseline characteristics and biochemical parameters

As shown in Table 1, aged rats (20 months old) presented significantly increased body and liver weight compared to 3‐month young rats, however, liver‐body weight ratio was moderately diminished in old rats. In addition, elderly animals exhibited certain decline in liver function as suggested by reductions in albumin levels and bile production, although no significant differences in transaminases (as a marker of liver cell injury) were observed. Evaluation of plasma lipids revealed an increase in cholesterol and LDL cholesterol, without significant changes in HDL cholesterol, triglycerides, and free fatty acids. Moreover, aged rats had higher hepatic lipid accumulation as compared to young rats by oil red O staining. Hepatic malondialdehyde (MDA) in old rats was elevated, suggesting an increase in oxidative stress secondary to lipid peroxidation process.

**Table 1:** Baseline characteristics and biochemical parameters of 3 months young and 20 months old rats

	3 months young	20 months old	% change	*p*‐value
Body weight (g)	380 ± 9	678 ± 22	+78	**<0.001**
Liver (g)	9.9 ± 0.3	15.3 ± 0.4	+54	**<0.001**
Liver‐body weight ratio (%)	2.6 ± 0.1	2.4 ± 0.1	−8	0.07
AST (U/L)	119.6 ± 12.2	113.2 ± 8.5	−5	>0.20
ALT (U/L)	48.9 ± 3.0	54.9 ± 6.1	+12	>0.20
Bile production (µl/min*100 g bw)	72.6 ± 6.8	31.7 ± 9.2	−56	**<0.001**
Albumin (mg/dl)	26.8 ± 0.5	24.9 ± 0.5	−7	**<0.001**
Plasma cholesterol (mg/dl)	52.1 ± 2.0	88.5 ± 8.8	+69	**<0.001**
Plasma LDL cholesterol (mg/dl)	24.3 ± 2.5	58.4 ± 5.4	+140	**<0.001**
Plasma HDL cholesterol (mg/dl)	16.3 ± 0.7	19.5 ± 2.2	+19	>0.20
Plasma triglycerides (mg/dl)	57.8 ± 9.1	53.5 ± 7.9	−7	>0.20
Plasma FFA (mg/dl)	662 ± 127	578 ± 99	−12	>0.20
Oil red O staining (%)	1.00 ± 0.18	5.31 ± 1.10	+431	**<0.001**
MDA (nmol/mg protein)	0.86 ± 0.23	2.70 ± 1.00	+213	0.09
LPS (EU/ml)	0.88 ± 0.05	1.28 ± 0.11	+45	**0.02**

Note

Data expressed as mean ± *SEM* (*n* = 12 each group)

AST: aspartate transaminase; ALT: alanine transaminase; FFA: free fatty acids; MDA: malondialdehyde; LPS: lipopolysaccharide.

As expected, analysis of senescence markers exhibited differences between young and old animals (Supporting Information Figure S1). P16, a protein involved in cell cycle regulation, was up‐regulated in liver tissue and in LSEC freshly isolated from aged rats. SIRT1, an enzyme related with longevity, was decreased in the aged liver. At last, we also observed hepatic telomere length attrition in old animals.

### 2.2 Aged animals present mild hepatic microcirculatory dysfunction

**Figure 1:**
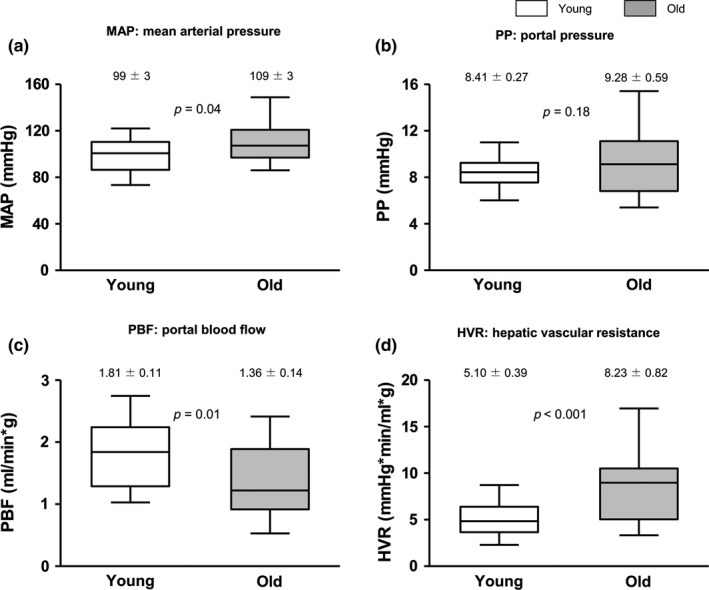
Hepatic and systemic hemodynamic of 3 months young and 20 months old rats. (a) Mean arterial pressure (MAP), (b) portal pressure (PP), (c) portal blood flow (PBF), and (d) hepatic vascular resistance (HVR) determined in 3 months young and 20 months old rats. *n* = 12 per group. Results represent mean ± *SEM*

When compared to young animals, old rats exhibited significantly higher hepatic vascular resistance (HVR) *in vivo*, associated with a reduction in liver perfusion, altogether leading to a moderately increased portal pressure (PP), without meeting criteria for portal hypertension (Figure 1). Mean arterial pressure was also significantly elevated, with no significant changes in heart rate (308 ± 9 vs. 319 ± 13 bpm).

*Ex vivo* perfusion experiments analyzing the liver endothelial‐dependent vasodilatory capacity showed no significant differences between groups (data not shown).

Mechanisms responsible of the observed age‐associated increased HVR were investigated in succeeding experiments.

### 2.3 Aging is associated with mild hepatocyte injury and dysfunction

Liver architecture was overall preserved in aged animals (Supporting Information Figure S2); however, evaluation of the severity of hepatic injury using a histological damage score revealed mild liver dysfunction in aged rats in comparison with young animals (Supporting Information Table S1). Indeed, injury analysis showed differences between groups in all of the evaluated parameters including cytoplasmic vacuolation, nuclear pyknosis, cytoplasmic hypereosinophilia, loss of intercellular borders, necrosis, neutrophil infiltration, and fat accumulation. Ultrastructural analysis of liver damage using transmission electron microscopy (TEM) evidenced a decrease in the number of sinusoids with no changes in other parameters. In addition, aged rats showed higher hepatic cell death as demonstrated by the TUNEL staining (Supporting Information Figure S3a), with no significant changes in apoptotic proteins c‐caspase‐3 and BAD (data not shown).

Liver tissue analyses were complemented examining the phenotype of hepatocytes freshly isolated from both groups of animals (Supporting Information Figure S3b–d). In agreement with the *in vivo* biochemical parameters, aged hepatocytes exhibited slight but not significant lower urea and albumin synthetic capacity. This was associated with deregulation in different specific markers including Oct1 and Mrp3, with no differences in Mrp2 or HNF4α. At last, hepatocyte cytochrome P4503A4 activity tended to be higher in aged animals.

### 2.4 The aged hepatic endothelium is *pseudocapillarized* and procontractile

**Figure 2:**
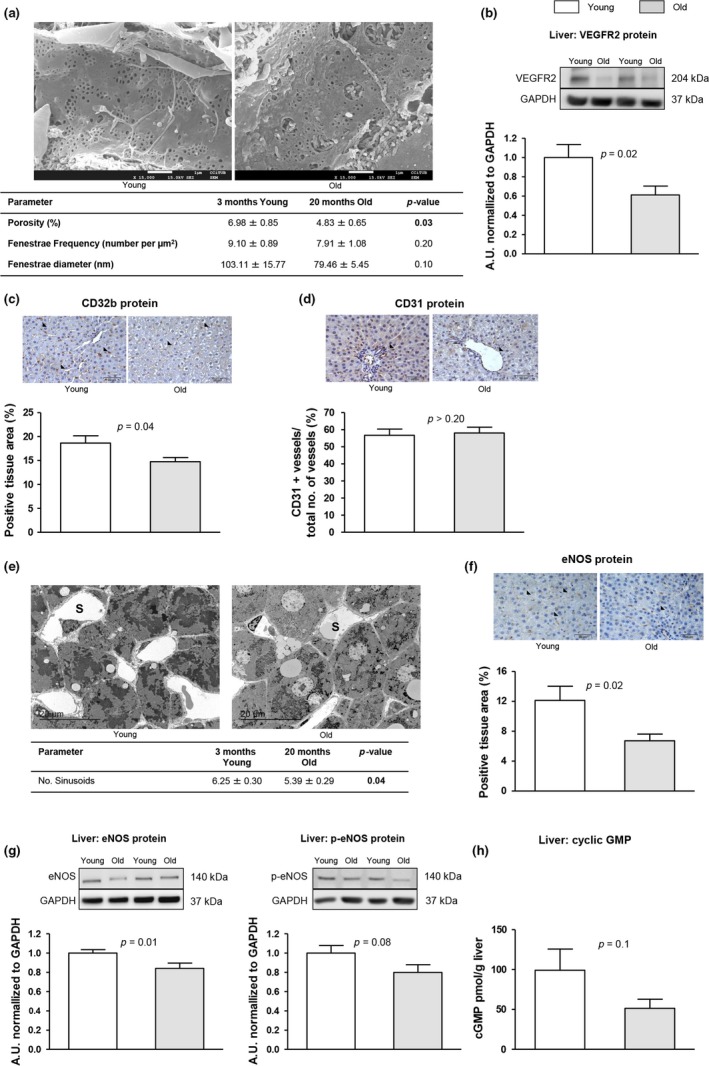
Endothelial dedifferentiation in the aged liver. The following markers of sinusoidal endothelial phenotype were analyzed in liver tissue from young and aged rats. (a) Representative scanning electron microscopy images and quantification of porosity, fenestration frequency, and fenestration diameter. (b) VEGFR2 protein expression normalized to GAPDH. (c) Representative images of CD32b immunohistochemistry and corresponding quantification. (d) Representative images of CD31 immunohistochemistry and corresponding quantification. (e) Representative transmission electron microscopy images and quantification of numbers of sinusoids. (f) Representative images of eNOS immunohistochemistry and corresponding quantification. (g) Representative western blots of eNOS and p‐eNOS, and protein quantification normalized to GAPDH. (h) Levels of cyclic GMP. *n* = 3 (a and e) and *n* = 12 (other panels) per group. Results represent mean ± *SEM*. All images 400x, scale bar=50 μm

Analysis of the liver sinusoid using scanning electron microscopy confirmed pseudocapillarization of aged LSEC in this experimental model of aging. As seen in Figure 2a, fenestrae porosity was markedly diminished in the aged hepatic sinusoid. In accordance, aged livers exhibited reduced expression of VEGFR2, a protein involved in fenestrae formation and maintenance, and CD32b, a well‐established marker of LSEC differentiation (Figure 2b,c).

Intrahepatic neovascularization analysis did not show differences between groups (Figure 2d). Nevertheless, the number of sinusoids seemed to be decreased in aged livers (Figure 2e), which correlates with the reduced CD32b expression described above.

Detrimental effects of aging on LSEC phenotype were not limited to markers of capillarization, but included loss of vasodilatory capacity. Indeed, aged livers presented reduced eNOS and p‐eNOS protein expression (Figure 2f,g), associated with a 45% reduction in the NO‐secondary messenger cGMP (Figure 2h).

**Figure 3:**
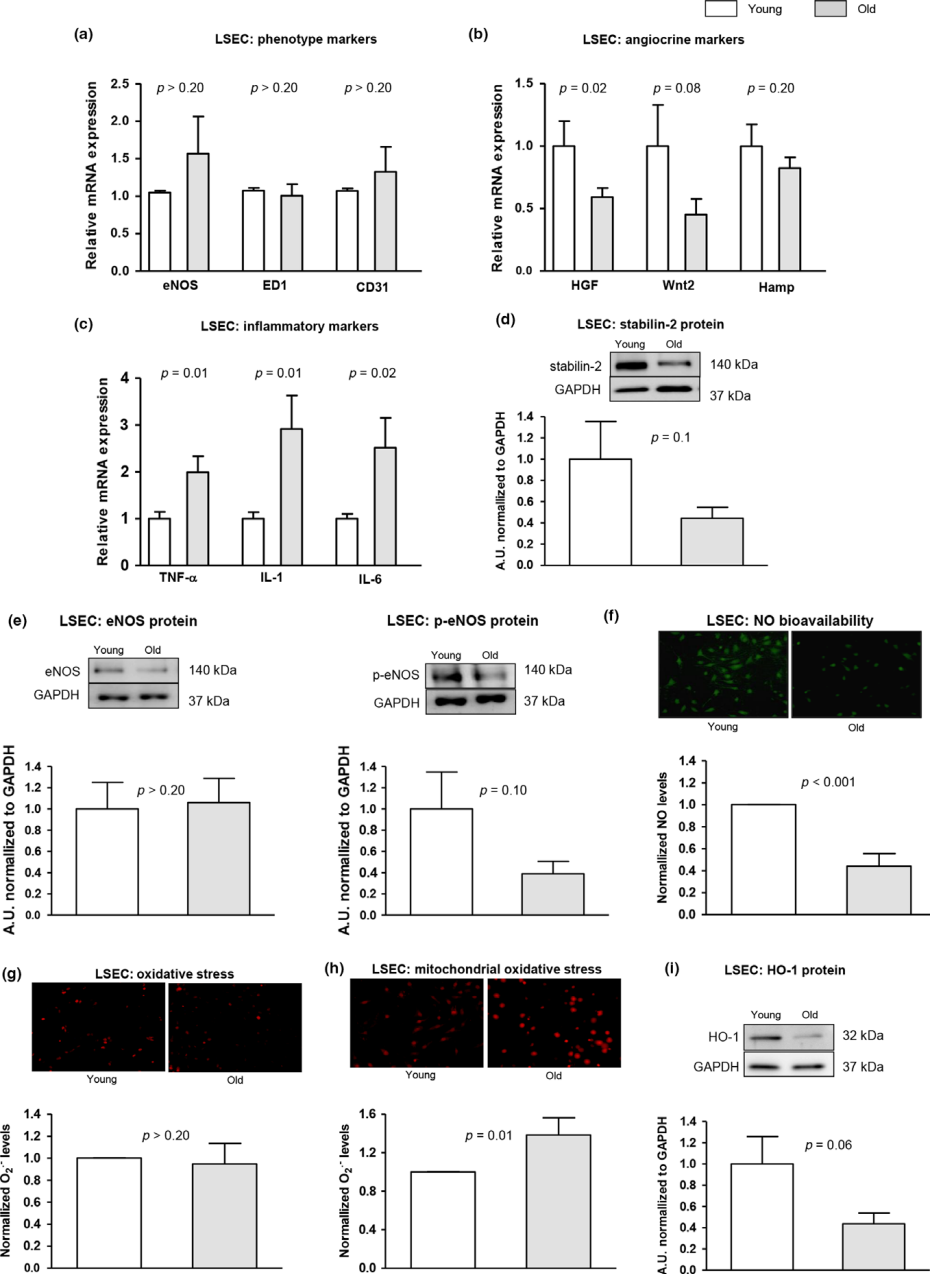
LSEC phenotype in aged livers. The phenotype of LSEC freshly isolated from aged and young rats was characterized as follows. (a) mRNA expression of eNOS, ED1, and CD31. (b) mRNA expression of HGF, Wnt2, and Hamp. (c) mRNA expression of TNF‐α, IL‐1, and IL‐6. (d) Representative western blot of stabilin‐2 normalized to GAPDH. (e) Representative western blots of eNOS and p‐eNOS, normalized to GAPDH. (f) Representative images of nitric oxide bioavailability using DAF‐FM staining (green fluorescence) and corresponding quantification. (g) Levels of O_2_^.^^‐^ (red fluorescence) determined using DHE. (h) Representative images of mitochondrial O_2_^.^^‐^ measured using mitosox (red fluorescence) and corresponding quantification. (i) HO‐1 protein expression normalized to GAPDH. *n* = 6 per group. Results represent mean ± *SEM*

Analysis of primary LSEC confirmed a remarkable dysfunction of cells isolated from aged animals (Figure 3). Although we did not observe significant changes in mRNA expression of eNOS, ED1, and CD31, aged LSEC showed alterations in angiocrine, inflammatory, scavenging, vasodilatory, and oxidative stress pathways. In fact, endothelial angiocrine molecules seemed to be decreased in aged LSEC, and the expression of different proinflammatory cytokines was up‐regulated. The scavenger receptor stabilin‐2 was decreased in aged LSEC. Moreover, p‐eNOS expression and NO bioavailability were reduced in aged LSEC in comparison with cells isolated from young animals. In addition, aged LSEC displayed significantly more mitochondrial oxidative stress, alongside reduced expression of the anti‐oxidant enzyme HO‐1.

### 2.5 Aged hepatic stellate cells are spontaneously activated

**Figure 4:**
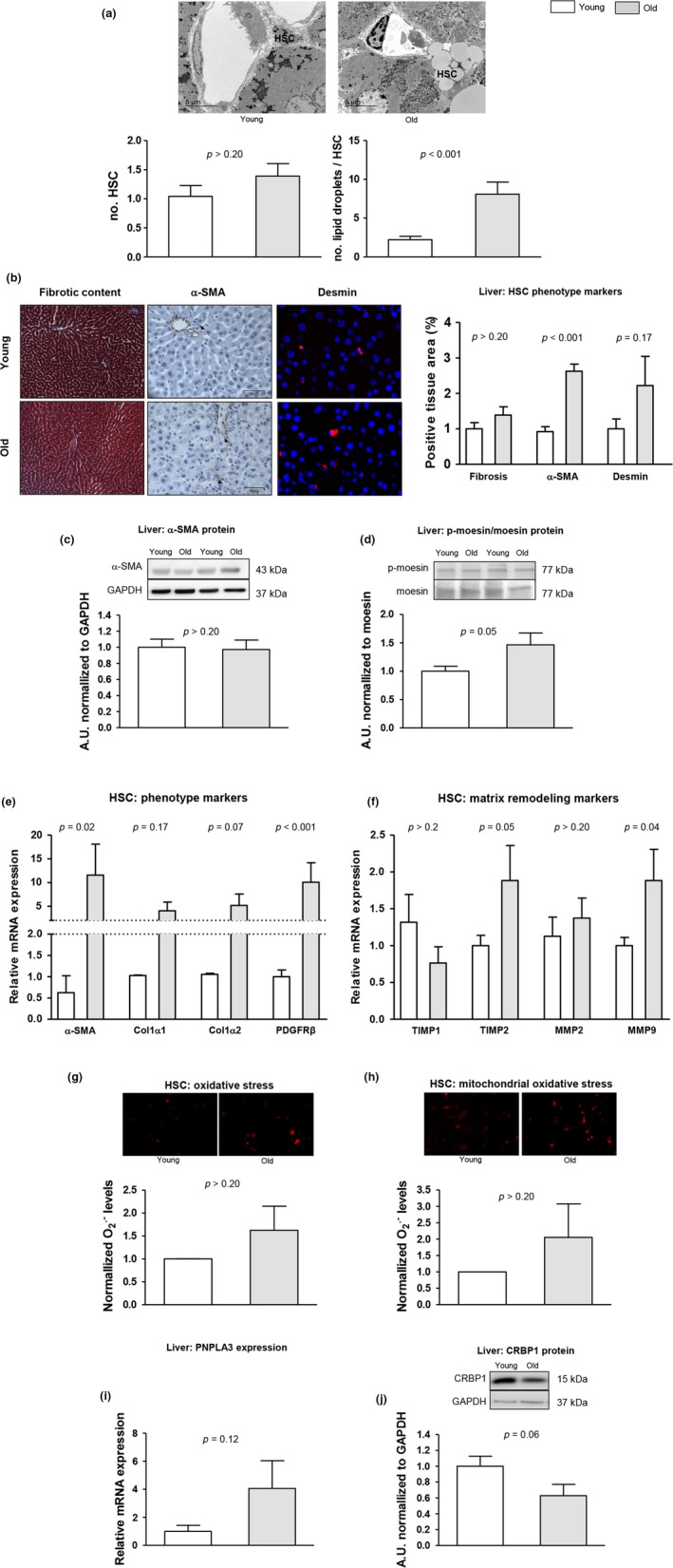
Effects of aging on HSC phenotype. The phenotype of HSC was characterized in aged and young rats as follows. (a) Quantification of HSC abundance and intracellular lipid droplets in livers using transmission electron microscopy. (b) Representative images of fibrotic content, measured as positive, are for Masson’s trichromic, α‐SMA, and desmin with their corresponding quantifications. (c) α‐SMA protein expression in total liver tissue, normalized to GAPDH. (d) p‐moesin protein expression in total liver tissue, normalized to total moesin. (e) Expression of α‐SMA, collagen1α1, collagen1α2, and PDGFRβ in freshly isolated HSC. (f) Expression of TIMP1, TIMP2, MMP2, and MMP9 in freshly isolated HSC. (g) Representative images of O_2_^.^^‐^ levels in primary HSC (red fluorescence) and corresponding quantification. (h) Mitochondrial O_2_^.^^‐^ levels (red fluorescence) determined in primary HSC. (i) Expression of PNPLA3 in total liver tissue. (j) CRBP1 protein expression in total liver tissue, normalized to GAPDH. *n* = 3 (a), *n* = 12 (b–d, i–j), and *n* = 6 (e–h) per group. Results represent mean ± *SEM*. All images 400×, scale bar = 50 μm

HSC phenotype was characterized both in liver tissue and freshly isolated cells (Figure 4). Analysis using TEM revealed that aged livers exhibit a nonsignificant increase in the number of stellate cells with higher number of intracellular lipid droplets in comparison with young. A trend for increased HSC in aged livers was supported by incremented desmin protein expression and significant increase in the proliferative HSC‐related growth factor PDGFRβ. In addition, aging was associated with slight, but significant, activation of HSC as demonstrated by increments in the mRNA and protein expression of different activation markers including α‐SMA, collagen1α1, collagen1α2, PDGFRβ, and p‐moesin, together with changes in some matrix remodeling genes including TIMP‐2 and MMP9. We observed a moderate but not significant increase in cellular and mitochondrial superoxide levels in aged HSC in comparison with young HSC. In an interesting manner, analysis of vitamin A metabolism pathways showed alterations in aged livers; indeed, PNPLA3 expression seemed to be increased while CRBP‐1 protein levels were diminished.

### 2.6 The aged hepatic sinusoid is in a moderate proinflammatory state

**Figure 5:**
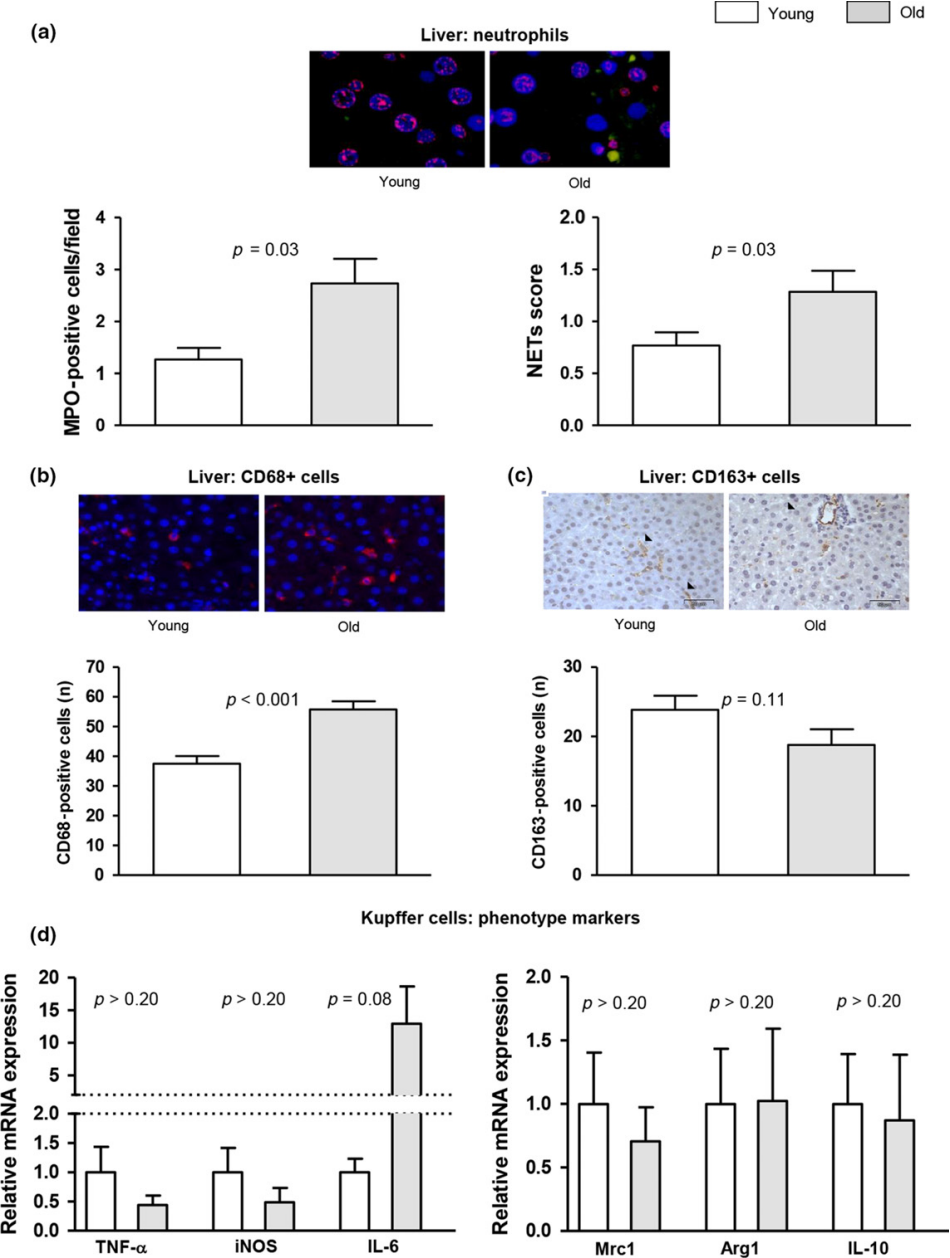
Innate immune cell activation and inflammation in aging. (a) Representative images of neutrophil immunofluorescence in liver tissue (MPO in green, histone 2B in red, nuclei in blue). *Left,* neutrophil infiltration measured as MPO‐positive cells. *Right,* analysis of hepatic neutrophil extracellular traps (NETs) determined as colocalization of MPO and histone 2B. (b) Representative images of CD68 immunofluorescence in liver tissue and corresponding quantification. (c) Representative images of CD163 immunohistochemistry in liver tissue and its quantification. (d) Expression of TNF‐α, iNOS, IL‐6, Mrc1, Arg1, and IL‐10 in Kupffer cells freshly isolated from young and old rats. *n* = 12 (a‐c) and *n* = 6 (d) per group. Results represent mean ± *SEM*. All images 400×, scale bar = 50 μm

Characterization of the inflammatory status of the liver evidenced that the aged hepatic sinusoid is rather proinflammatory as suggested by elevated presence and activity of infiltrated myeloid cells including neutrophils, increased amount of CD68‐positive macrophages, and diminished presence of CD163‐positive cells (Figure 5a–c).

When we further analyzed the phenotype of freshly isolated aged KC, we observed an increase in the inflammatory cytokine IL‐6, a key driver of liver regeneration, infection defense, and regulation of metabolic functions (Wuestefeld et al., 2013) without changes in the mRNA expression of other inflammatory molecules (Figure 5d).

### 2.7 Bacterial translocation and intestinal inflammation are not affected in elderly rats

In comparison with young rats, aged animals did not present discrepancies in gut bacterial translocation to mesenteric lymph nodes, feces bacterial load, nor in the expression of the cytokines IFN‐γ, TNFα, and IL17α measured in the ileum (data not shown). In contrast, a minor increase in LPS plasmatic levels was observed in aged rats (Table 1).

### 2.8 The aged human liver displays similar features of a dysfunctional sinusoid

**Figure 6:**
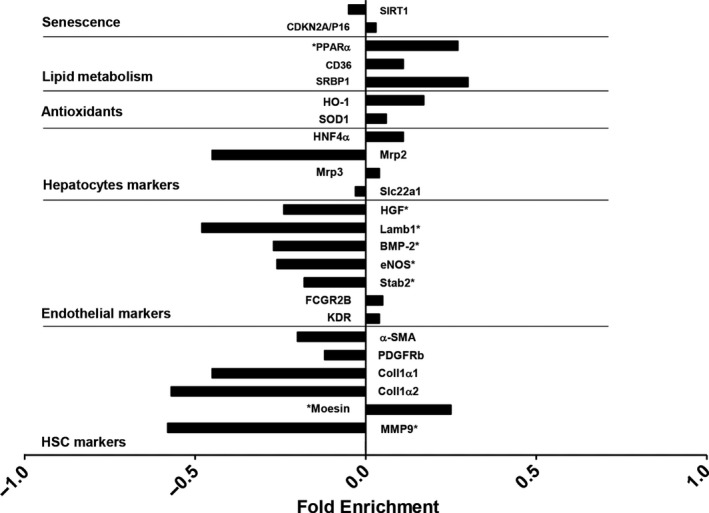
Aged‐related changes in the human liver. Gene expression analysis in healthy young and old human livers. Data expressed as fold enrichment (log_2_) old relative to young (0.0). The fold enrichments are plotted using positive values for transcripts that are increased or using negative values for transcripts that are decreased in old humans. *p‐value <0.05, *n* = 14 young and 13 old livers. Clinical characteristics of donors are described in Supporting Information Table S2

Characterization of the liver sinusoid in young and old human tissues corroborated most of the molecular changes observed in the animal model (Figure 6). Indeed, aged livers exhibited features of LSEC de‐differentiation as suggested by significant reductions in angiocrine and vasodilatory gene expression levels, together with partial activation of HSC (Supporting Information Figure S4). As expected, lipid metabolism and senescence pathways were increased in aged patients.

## 3 DISCUSSION

The socioeconomic and medical care improvements occurring during the last decades have led to a relevant increase in the elderly, especially in developed countries (He et al., 2016). Therefore, it is critical to understand the molecular basis of aging and to identify possible approaches for therapeutic intervention in case major abnormalities are detected (Longo et al., 2015; Mitchell, Morten, Longo, & Cabo, 2015). Although previous studies described the impact of aging on the vasculature of different territories, in the specific field of Hepatology, very little is known about the liver microcirculatory function and the molecular status of hepatic sinusoidal cells in aging (Le Couteur & McLean, 1998; Le Couteur et al., 2001).

In the present study, we demonstrate for the first time that healthy aged rats exhibit a significant increase in the hepatic vascular resistance, which leads to reduced liver perfusion and to a mo‐derate increment in portal pressure. Although a reduction in portal blood inflow was previously reported in humans and rodents (Vollmar et al., 2002), we herein describe a deregulation in the intra‐hepatic vascular system due to diverse molecular changes in the cells of the hepatic sinusoid, together with deterioration in hepatocyte function.

Analysis of hepatocytes revealed mild liver dysfunction in aged animals, which could likely be a consequence of altered oxygen and nutrient supply following the alterations in the vascular system. We observed a reduction in the liver‐body weight ratio, which was accompanied by a significant decline in synthetic and detoxifying capabilities as demonstrated by reduced bile formation and decreased urea and albumin synthesis, together with dysfunctional expression of hepatocyte transporters. These findings agree with previous reports (Le Couteur & McLean, 1998; López‐Otín, Blasco, Partridge, Serrano, & Kroemer, 2013; Tietz, Shuey, & Wekstein, 1992), altogether suggesting a decrease both in the amount and functional capacity of hepatocytes in aging, therefore advising a potential enhanced susceptibility to secondary insults such as ischemia/reperfusion or drug‐induced liver injury. From a clinical perspective, it is important to note that these events are especially common in elderly population, which have the higher prevalence of circulatory diseases and receive multiple medications with potential hepatotoxicity.

Underlying mechanisms explaining hepatocyte dysfunction may relate to adaptations following vascular dysfunction, to other age‐derived intracellular deregulation, or to cell senescence per se. Senescence, a cellular mechanism imposed by natural exhaustion of cell cycle or by stress‐induced signals, was evident in aged animals as demonstrated by up‐regulation in p16 along with a reduction in telomere length and SIRT1 protein. In addition, we observed an increase in hepatic oxidative stress, with multiple harmful consequences, that may derive from an imbalance between pro‐ and anti‐oxidants, together with increased hepatic lipid content, a well‐known source of cellular oxidative stress. In an important way, oxidative stress could also contribute to potentiate the senescence pathways, and vice versa, thus creating a deleterious vicious cycle in the aged liver (Jin, Iakova, Jiang, Medrano, & Timchenko, 2011; Sharpless & De Pinho, 2004).

Characterization of liver microcirculatory dysfunction in aged animals confirmed LSEC “pseudo‐capillarization,” which was defined by the decrease in the number and size of fenestrae (Le Couteur et al., 2001). Further analysis revealed a global deregulation in the hepatic endothelial phenotype in aging as demonstrated by significant decrease in key vasodilatory pathways, including nitric oxide and heme oxygenase, increment in intracellular inflammation and oxidative stress, and reduction in the expression of functional and angiocrine markers such as stabilin‐2, CD32b (Xie et al., 2013), and VEGFR2 (Carpenter et al., 2005). Reduction in intrahepatic nitric oxide availability is of relevance considering its importance modulating the vascular tone (Gracia‐Sancho, Maeso‐Diaz, Fernandez‐Iglesias, Navarro‐Zornoza, & Bosch, 2015; Hori, Wiest, & Groszmann, 1998), exerting anti‐inflammatory effects (Iwakiri & Kim, 2015), and maintaining neighboring cells phenotype (Marrone et al., 2016; Xie et al., 2013). Reduced nitric oxide bioavailability might, at least in part, derive from diminished eNOS activity, which may be due to reduced VEGF‐p‐eNOS pathway (Kroll & Waltenberger, 1998), and from increased scavenging due to elevated oxidative stress (Gracia‐Sancho et al., 2008). An additional mechanism contributing to VEGF‐p‐eNOS pathway depletion may be the increase in the senescence marker p16, which exerts an inhibitory effect on VEGF (Kawagishi et al., 2010). Nevertheless, a previous study reported no changes in hepatic VEGF expression in aging (Le Couteur et al., 2007). Therefore, this issue would require further investigation to confirm the current findings.

Similar to nitric oxide, the observed diminution in HO‐1 expression in the aged endothelium may contribute to the increased HVR (Van Landeghem et al., 2009). Altogether our results strongly suggest that vasodilatory pathways in LSEC are profoundly affected by age and this might be one of the mechanisms responsible of aged‐associated increase in HVR.

A previous report described increased number of fatty HSC in the aged liver (Warren et al., 2011); however, no comprehensive analysis of stellate cells phenotype in aging has been conducted. Herein, we confirm lipid accumulation within aged HSC and additionally describe increments in other mediators of HSC activation including intracellular oxidative stress and p16 (Bataller et al., 2003). In fact, analysis of cellular phenotype revealed a slight, but significant, activation of HSC, supported by increments in different activation markers including α‐SMA or collagen I. Underlying mechanisms explaining HSC activation in aging revealed no modification in different proinflammatory mediators including TLR4, NFκB, and TGFβ (data not shown), but interestingly suggested alterations in retinoid metabolism and breakdown. In fact, aged livers exhibited down‐regulated CRBP‐1 together with over‐expressed PNPLA3, which have been associated with HSC activation and susceptibility to develop steatohepatitis (Bruschi et al., 2017; Uchio et al., 2002).

Very few data regarding inflammation and KC phenotype in aging are available, and the data are inconclusive. For instance, while one study reported that KC lose their phagocytic capacity with aging (Brouwer & Knook, 1983), a subsequent study reported increased phagocytic activity in KC from aged animals (Hilmer, Cogger, & Le Couteur, D. G., 2007). In our evaluation, we noticed an increase in the recruitment of proinflammatory cells with a concomitant increment in inflammatory markers, altogether suggesting that aging may promote a moderate proinflammatory status in the liver. Kupffer cells isolated from aged rats displayed high IL‐6 levels, which could possibly reflect beneficial effects of IL‐6 on liver regeneration, infection defense, and regulation of metabolic functions (Schmidt‐Arras & Rose‐John, 2016), while traditional “polarization markers” of hepatic macrophages (Krenkel & Tacke, 2017) were not dramatically altered. However, given the complexity of inflammation and immune cell subsets, further investigations are required to define, which inflammatory processes are functionally relevant during aging.

The translocation of bacterial products from the gut is the consequence of intestinal barrier disruption and may lead to bacterial infection and have consequences in terms of liver and systemic inflammation and HSC activation. Considering the hemodynamic and inflammatory changes observed in aged animals, we aimed at analy‐zing bacterial translocation, fecal bacterial load, intestinal inflammation, and endotoxemia in old rats. In an interesting manner, these analyses revealed no significant differences in most of these parameters when comparing both groups of rats, except for LPS plasmatic levels that were increased in aged rats. We therefore cannot discard that the observed minimal aged‐related endotoxemia may contribute to the abnormalities of sinusoidal cells.

At last, and although an undeniable degree of heterogeneity was found in each group analyzed, characterization of liver biopsies from healthy young and old humans confirmed the overall trend of pathway deregulation, therefore suggesting that sinusoidal vulnerability hereby described in old rats is also of relevance in human aging.

Many efforts are currently being directed toward the development of novel therapeutic approaches for portal hypertension (Gracia‐Sancho et al., 2015). Most preclinical studies on the effects of different substances and drugs on the liver circulation have been conducted in young animals. Our current work revealed striking differences between young and aged liver, including alterations in vasoactive pathways, indicating that studies performed in young animals may not entirely reflect what should be expected from the cli‐nical use of most interventions, as vasoactive drugs are and will be mainly prescribed in the elderly.

We are aware that our study is descriptive in nature. However, this work represents an important cornerstone characterization of liver aging. While single variables have been examined previously, no preceding report fully described the liver microcirculatory phenotype in a preclinical model of healthy aging, additionally including validation in human samples. These data provide important evidence for the changing biology of the liver in aging and create new research avenues to comprehend the effects of acute or chronic liver injury in the elderly. It is important to acknowledge that aged animals exhibited some degree of steatosis; therefore, part of the results could be attributable to intracellular fat instead of age. Nevertheless, a subanalysis of animals without evidence of significant steatosis confirmed the same hemodynamic and molecular results than in the group of animals exhibiting steatosis (data not shown), therefore ensuring that the results of this study describe the effects of aging per se on liver microcirculatory phenotype.

In conclusion, the present study demonstrates for the first time that aging is accompanied by significant liver sinusoidal deregulation, both in rodents and humans, suggesting sinusoidal vulnerability in front of subsequent chronic or acute injuries.

## 4 EXPERIMENTAL PROCEDURES

A complete description of Materials and Methods can be found in Appendix S1.

### 4.1 Animal model

Male Wistar rats at the age of 20 months old were used to evaluate aging and compared to young animals of 3 months old (*n* = 12 animals per group) (Steppan et al., 2014; Wang, Wehling‐Henricks, Samengo, & Tidball, 2015). Animals were kept in environmentally controlled animal facilities at IDIBAPS. All procedures were approved by the Laboratory Animal Care and Use Committee of the University of Barcelona and were conducted in accordance with the European Community guidelines for the protection of animals used for experimental and other scientific purposes (EEC Directive 86/609).

### 4.2 *In vivo* hemodynamic measurements

Mean arterial pressure (MAP), portal pressure (PP), and portal blood flow (PBF) were measured in old and young rats using microcatheters and flow probes (Marrone et al., 2015). Hepatic vascular resistance (HVR) was calculated as PP/PBF.

### 4.3 Hepatic cells isolation

Hepatocytes, Kupffer cells (KC), liver sinusoidal endothelial cells (LSEC), and hepatic stellate cells (HSC) were isolated using well‐established protocols (De Mesquita et al., 2017; Gracia‐Sancho et al., 2007). Only highly pure and viable cells were used.

### 4.4 Electron microscopy

Liver sinusoidal ultrastructure was characterized using electron microscopy as previously described (Le Couteur et al., 2001).

### 4.5 Histological analysis

Liver samples were fixed in 10% formalin, embedded in paraffin, sectioned, and slides were stained with hematoxylin and eosin (H&E) to analyze the hepatic parenchyma (Hide et al. 2016), with Masson’s trichrome for liver fibrosis evaluation (Gracia‐Sancho et al., 2011), with oil red O for lipid quantification or with corresponding antibodies for immunohistochemistry (IHC) or immunofluorescence (IF; Marrone et al., 2015).

### 4.6 Cell death

Terminal deoxynucleotidyl transferase dUTP nick end labeling (TUNEL) was performed in deparaffinized liver sections using an In Situ Cell Death Detection Kit (Roche Diagnostics, Sant Cugat del Valles, Barcelona, Spain) according to the manufacturer’s instructions (Hide et al., 2014).

### 4.7 Nitric oxide and superoxide determinations

Levels of cGMP, marker of nitric oxide bioavailability, were analyzed in liver homogenates using an enzyme immunoassay following manufacturer instructions (Cayman Chemical Co., Ann Arbor, MI) (Gracia‐Sancho et al., 2008). In situ superoxide and nitric oxide levels in cells were assessed with the oxidative fluorescent dye dihydroethidium (DHE 10 µM; Molecular Probes Inc., Eugene, OR) or with 4‐amino‐5‐methylamino‐2′,7′‐difluorofluorescein diacetate (DAF‐FM‐DA 10 µM; Molecular Probes Inc.), respectively, as described (Hide et al., 2014). Fluorescence images were obtained with a fluorescence microscope (Olympus BX51, Tokyo, Japan), and quantitative analysis of at least 20 images per condition containing equivalent number of cells was performed with Image J 1.44m software.

### 4.8 Human liver samples collection for mRNA analysis

Two‐by‐two cm wedge sharp cuts were obtained from the liver from donors after brain death without the use of electrocautery, once the donor next of kin provided written informed consent (Document R‐DON‐049 02/15). Biopsies were immediately stored in diethyl pyrocarbonate solution, a nuclease inhibitor, for mRNA analysis. Ethics Committee of the Barcelona Hospital Clinic approved the experimental protocol (HCB/2011/6499). RNA extraction and gene expression analysis were performed as described in Supporting Information Appendix S1 (Experimental Procedures). Experimental groups were defined considering donors’ age: young (*n* = 14, mean age 28 ± 2 years old, range 19–38) and old (*n* = 13, mean age 76 ± 0.8, range 71–85). No differences in other clinical parameters were found comparing groups (Supporting Information Table S2).

### 4.9 Statistical analysis

Statistical analysis was performed with the SPSS for Windows statistical package (IBM, Armonk, New York, USA). All results are expressed as mean ± standard error of the mean (*SEM*). Comparisons between groups were performed with Student's *t* test. Differences were considered significant at a *p* value <0.05.

## CONFLICT OF INTEREST

Authors have no conflict of interest.

## AUTHORS CONTRIBUTIONS

R.M.‐D. designed the research, performed experiments, analyzed data, and wrote the manuscript. M.O.‐R., A.F.‐I., D.H., L.M., S.V., and R.F. performed experiments and analyzed data. A.H. and C.F. procured human liver tissue for the study, interpreted data and critically revised the manuscript. A.A., C.P., J.B., F.T., and V.C. interpreted data and critically revised the manuscript. J.G.‐S. conceived the study, designed and directed the research, analyzed and interpreted data, wrote the manuscript, and obtained funding. All authors edited and approved the final manuscript.
