# Supplementary figures and images for: Effects of aging on liver microcirculatory function and sinusoidal phenotype
Source: Aging Cell. 2018 Sep 8;17(6):e12829. doi: 10.1111/acel.12829 (PMC6260924; doi:10.1111/acel.12829)

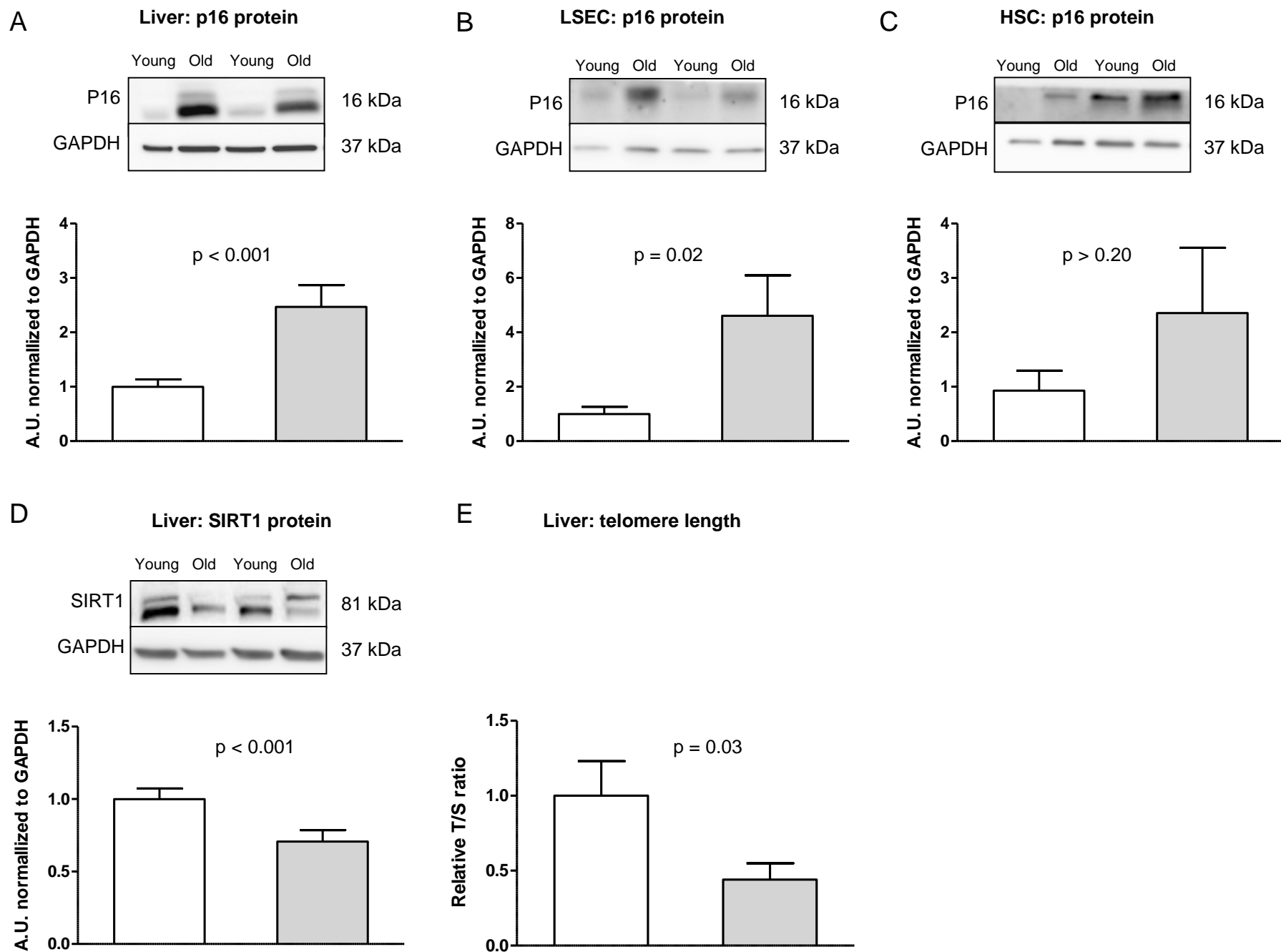

Supplement: Supplementary file 1 [file ACEL-17-e12829-s001.pdf]

**Liver architecture**

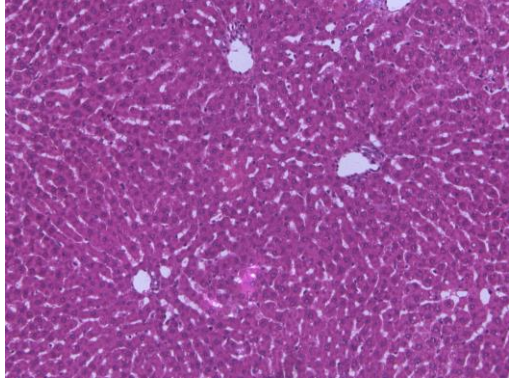

Young

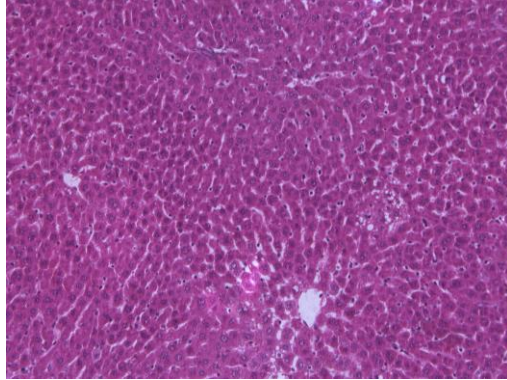

Old

Supplement: Supplementary file 2 [file ACEL-17-e12829-s002.pdf]

Young Old

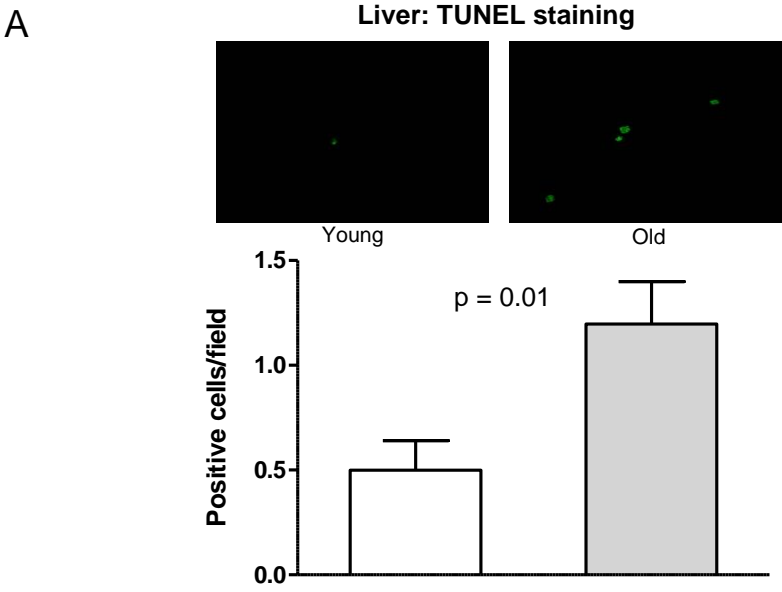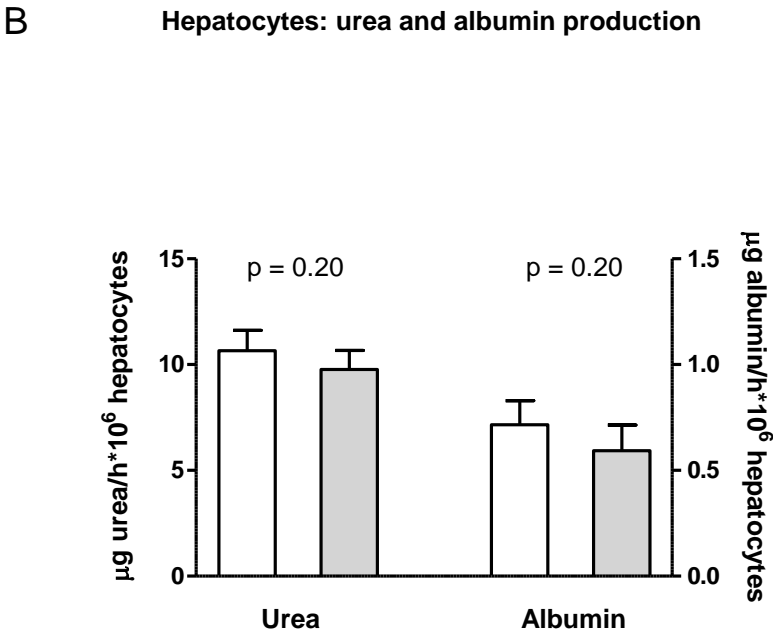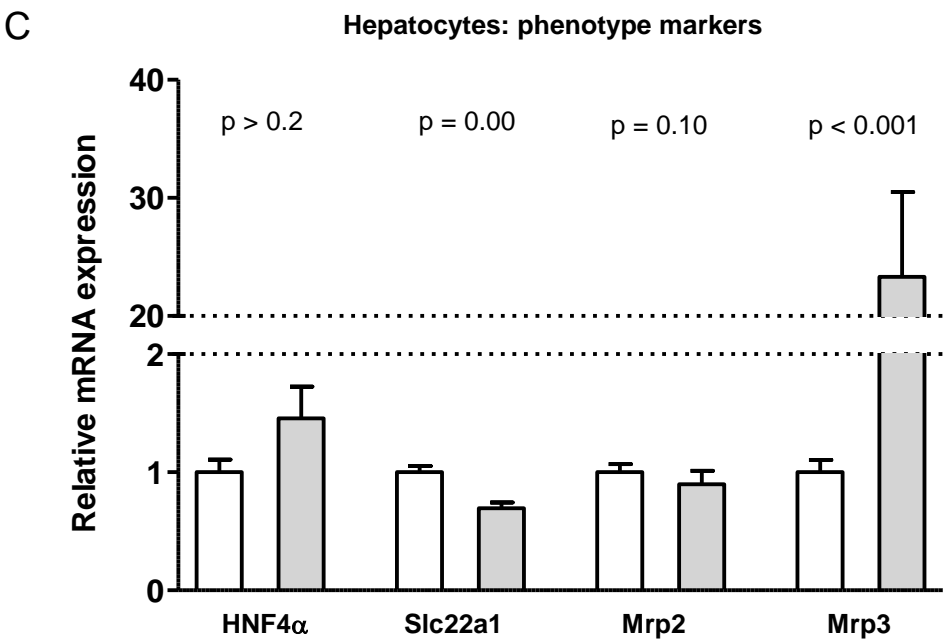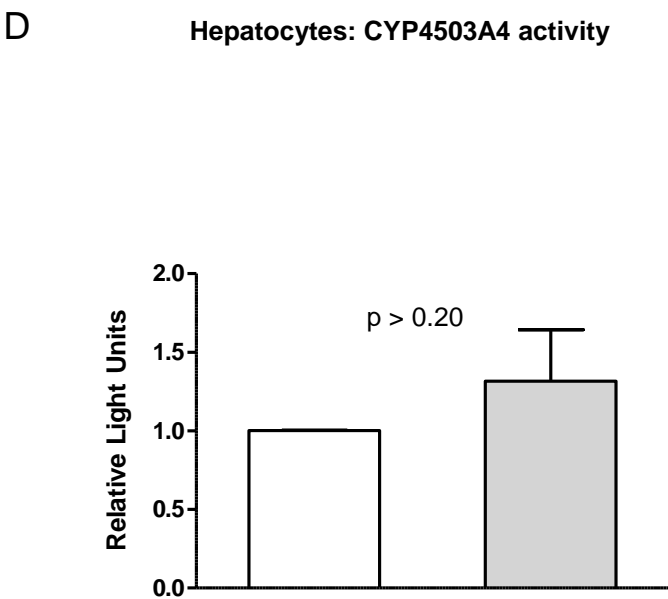

Supplement: Supplementary file 3 [file ACEL-17-e12829-s003.pdf]

Young Old

A

Liver: Desmin staining

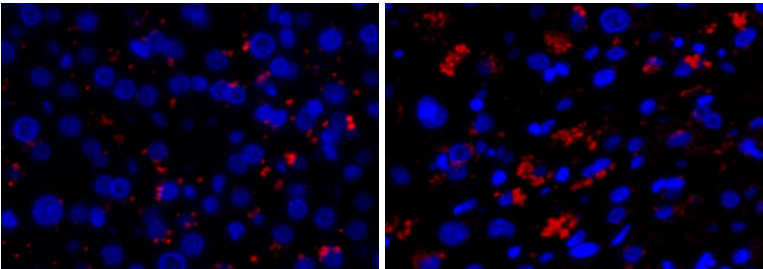

Young

Old

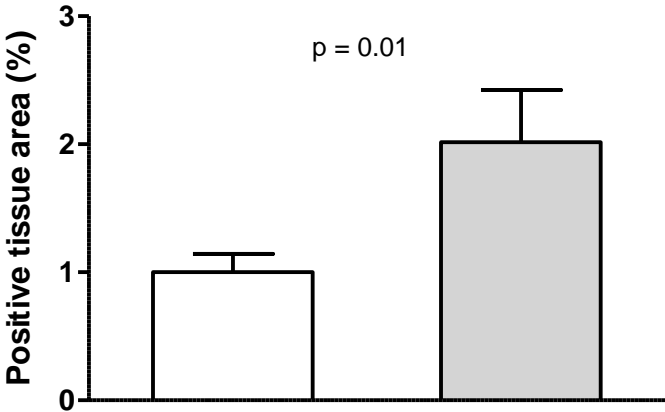

B

Liver:  $\alpha$ -SMA staining

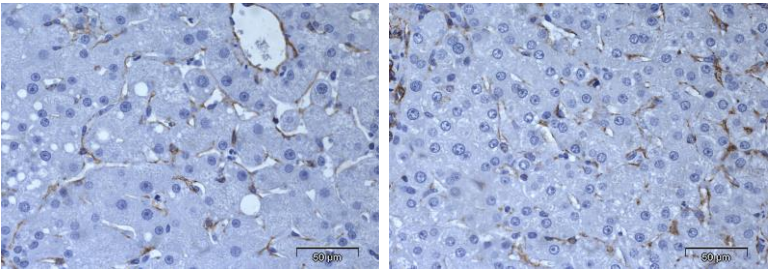

Young

Old

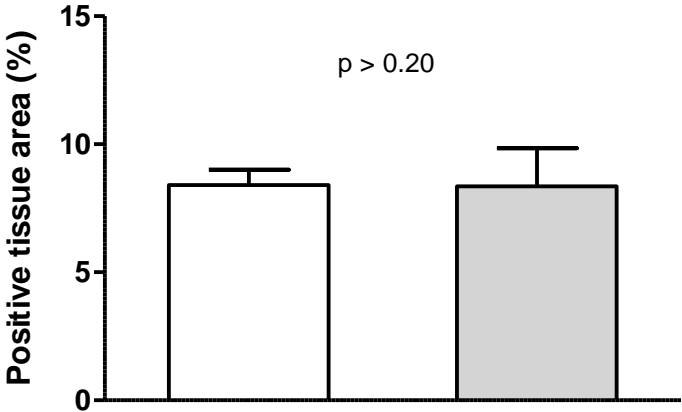

Supplement: Supplementary file 4 [file ACEL-17-e12829-s004.pdf]

Total hepatic tissue western blot

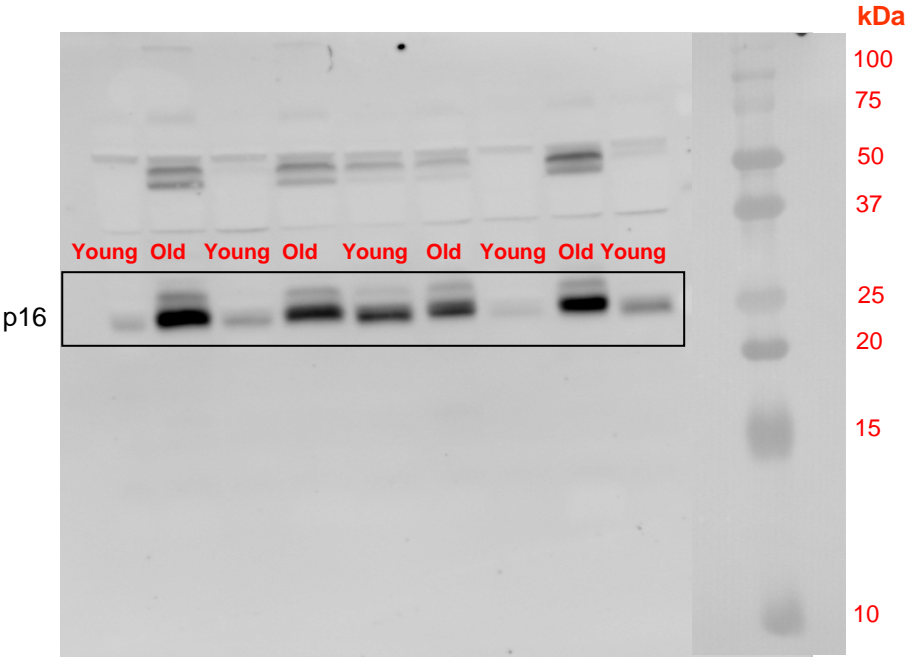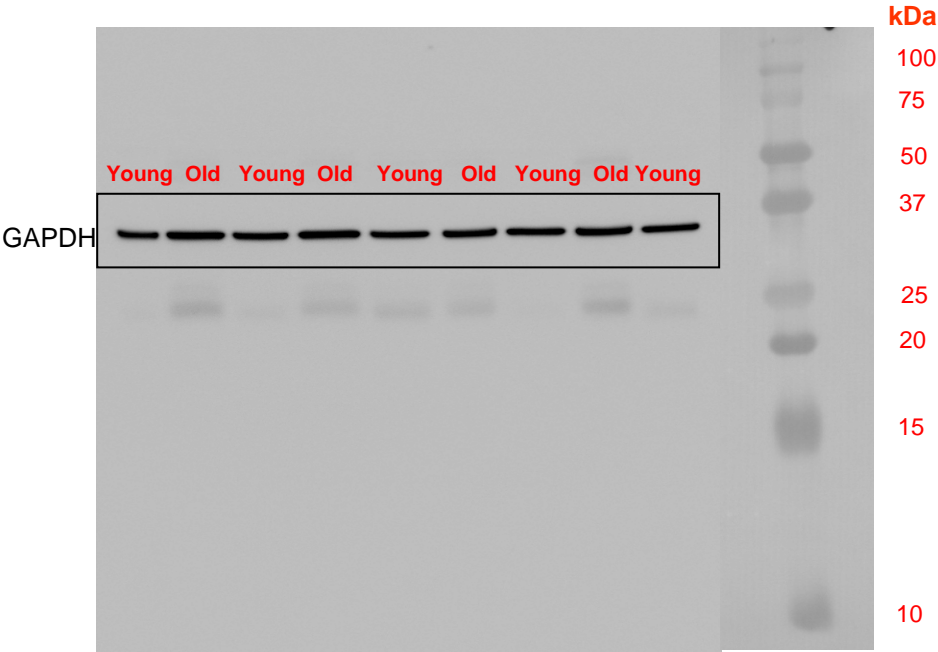

LSEC

kDa

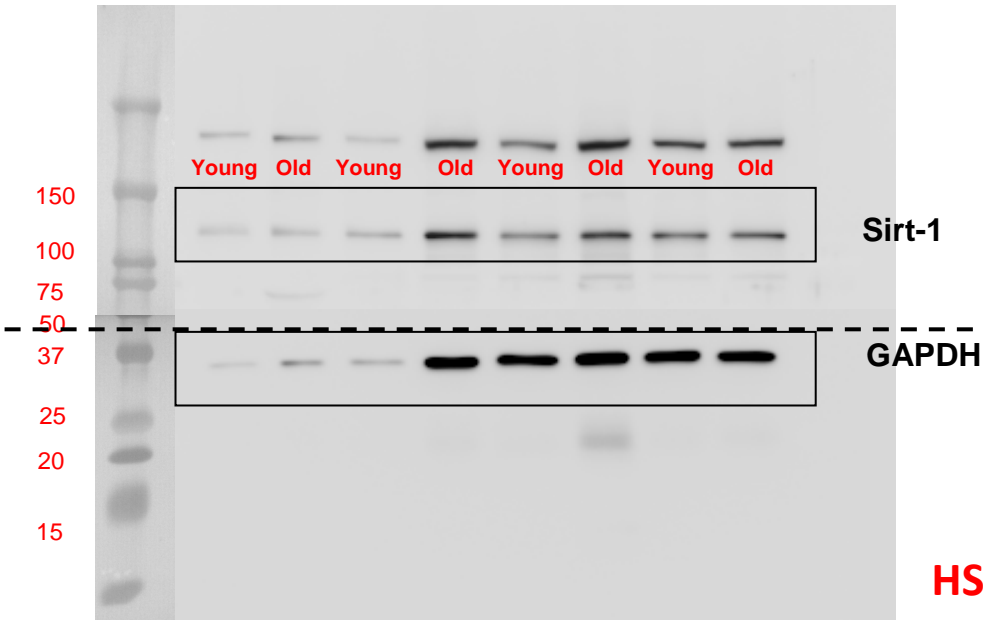

HSC

kDa

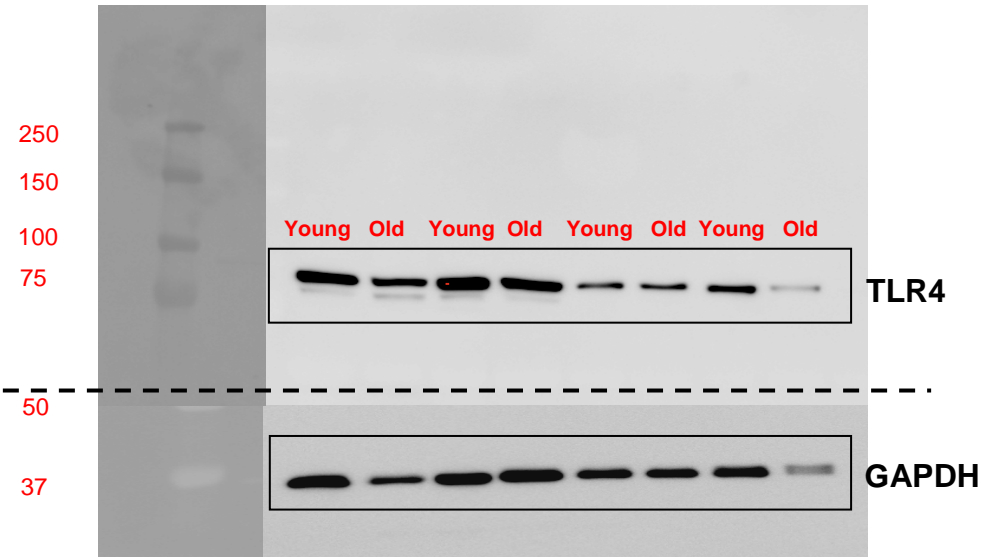

Supplement: Supplementary file 6 [file ACEL-17-e12829-s006.pdf]
