# Supplementary material for: Effects of aging on liver microcirculatory function and sinusoidal phenotype
Source: Aging Cell. 2018 Sep 8;17(6):e12829. doi: 10.1111/acel.12829 (PMC6260924; doi:10.1111/acel.12829)
Supplement: Supplementary file 5 [file ACEL-17-e12829-s005.docx]

**Effects of aging on liver microcirculatory function and sinusoidal phenotype**

Raquel Maeso-Díaz, Martí Ortega-Ribera, Anabel Fernández-Iglesias, Diana Hide, Leticia Muñoz, Ammelia Hessheimer, Sergi Vila, Rubén Francés, Constantino Fondevila, Agustín Albillos, Carmen Peralta, Jaime Bosch, Frank Tacke, Victoria C. Cogger, Jordi Gracia-Sancho

**Supplementary Experimental Procedures**

**In vivo hemodynamic**

Rats were anesthetized with ketamine (100 mg/kg body weight, Imalgene 1000; Merial) plus midazolam (5 mg/kg body weight; Laboratorio Reig Jofre, S.A., Spain) intraperitoneally, fastened to a surgical board, and maintained at a constant temperature of 37 ± 0.5ºC.

A tracheotomy and cannulation with a PE-240 catheter (Portex) was performed in order to maintain adequate respiration during anesthesia. Indwelling catheters made of polyethylene tubing (PE-50; Portex, UK) were placed into the femoral artery to measure mean arterial pressure (MAP; mm Hg) and heart rate (HR; beats per minute), and to the ileocolic vein to measure PP (mmHg). PBF (mL/min) was measured with a nonconstrictive perivascular ultrasonic transit-time flow probe (2PR, 2-mm diameter; Transonic Systems Inc., USA) placed around the portal vein just before its entrance in the liver, avoiding the measurement of most portal-collateral blood flow. The flow probe and pressure transducers were connected to a Powerlab (4SP) linked to a computer using Chart v5.5.6 for Windows software (AD Instruments, Australia). Hepatic vascular resistance (HVR) was calculated as PP/PBF. Hemodynamic data were collected after a 20-minute stabilization period (Gracia-Sancho et al. 2007). At the end of the in vivo hemodynamic study, serum samples from young and aged-rats were collected to subsequently evaluate alanine aminotransferase (ALT), aspartate aminotransferase (AST), bilirubin, and albumin, all by standard protocols. Experiments and data collection were performed blindly.

**Hepatic cells isolation**

Hepatocytes, Kupffer cells (KC) and Liver Sinusoidal Endothelial Cells (LSEC) were isolated using a well established protocol (Gracia-Sancho et al. 2007). Rat livers were perfused through the portal vein with Hanks without Ca^+2^ and Mg^+2^ containing 12 mM hepes (H3375, Sigma) pH 7.4, 0.6 mM ethylene glycol-bis (2-aminoethylether)-N,N,N′,N′-tetraacetic acid (E4378, Sigma) and 0.23 mM bovine serum albumin (BSA; A1391,0100, Applichem). Then, perfused for 30 min with 0.015% collagenase A (103586, Roche) Hanks containing 12 mM hepes (pH 7.4) and 4 mM CaCl_2_. The resultant digested liver was excised and *in vitro* digestion was performed at 37°C with 0.01% collagenase A, Hank’s containing 12 mM hepes (pH 7.4) and 4 mM CaCl_2_ for 10 min. Disaggregated tissue was filtered using 100 µm nylon strainer, collected in cold Krebs’ buffer and centrifuged at 50 g for 5 min. The pellet was washed three times for hepatocytes enrichment. Hepatocytes were seeded in collagen-coated wells and cultured in Dulbecco’s Modified Eagle’s Medium (DMEMF12; 11320074, Gibco) supplemented with 2% fetal bovine serum (04-001-1A, Reactiva), 1% L-glutamine (25030-024, Gibco), 1% penicillin plus 1% streptomycin (03-331-1C, Reactiva), 1 nM dexamethasone (D4902, Sigma), 1 μM insulin (103755, HCB) and 1% amphotericin B (03-029-1C, Reactiva). The supernatant was centrifuged at 800g for 10 min and the obtained pellet was resuspended in Dulbecco’s PBS (DPBS) and centrifuged at 800g through a two-step Percoll gradient (25-50%). The interface of the gradient was enriched in KC and LSEC. This cell fraction was diluted in DPBS and centrifuged at 800g. The cell pellet was resuspended in RPMI medium, seeded in plastic dishes and incubated for 30 min at 37ºC in humid atmosphere with 5% CO_2_ in order to enhance KC purity. Non-adherent cells were seeded in collagen-coated wells and incubated for 1h (37ºC, 5% CO_2_). After this time the medium was discarded and LSEC adhered cells were washed twice with DPBS and cultured in RPMI-1640 (01-100-1A, Reactiva) supplemented with 10% fetal bovine serum (04-001-1A, Reactiva), 1% L-glutamine (25030-024, Gibco), 1% penicillin plus 1% streptomycin (03-331-1C, Reactiva), 0.1 mg/ml heparin (H3393, Sigma), 0.05 mg/ml endothelial cell growth supplement (BT-203, BT) and 1% amphotericin B (03-029-1C, Reactiva). HSC were isolated through a sequential in situ perfusion of the liver with 0.195 mg/ml collagenase A (Roche), 1.5 mg/ml pronase (Roche) and 0.05 mg/ml Dnase (Roche) in Gey’s Balanced Salt Solution (GBSS; Sigma), and dispersed cells were fractionated by density gradient centrifugation using 11.5% Optiprep (Sigma) (De Mesquita et al. 2017). HSC were cultured in Iscove’s Modified Dulbecco’s Media (IMDM, Invitrogen, Gibco) supplemented with 10% fetal bovine serum (04-001-1A, Reactiva), 1% L-glutamine (25030-024, Gibco), 1% penicillin plus 1% streptomycin (03-331-1C, Reactiva) and 1% amphotericin B (03-029-1C, Reactiva). Viability and purity were systematically over 95%.

**Electron microscopy**

Liver sinusoidal ultrastructure was characterized using electron microscopy as previously described (Le Couteur et al. 2001). Briefly, livers were perfused through the portal vein with a fixation solution containing 2.5% glutaraldehyde and 2% paraformaldehyde in 0.1M cacodylate buffer 0.1% sucrose and fixed overnight at 4ºC. Samples were washed 3 times with 0.1M sodium cacodylate buffer. Liver sections were post-fixed with 1% osmium in cacodylate buffer and dehydrated in an ethanol gradient to 100%.

For scanning electron microscopy 6 to 8 liver blocks per sample were mounted on stubs, sputter coated with gold and examined using a Jeol 6380 scanning electron microscope. Measurements of fenestrae size, number and density were carried out. Fenestrations were defined as open pores with diameters <300nm. Diameter was defined as the major length of each fenestration or gap. Porosity was defined as the sum area of fenestrations/total quantified area. Frequency was defined as number of fenestrae per μm^2^. At least 10 images per animal were taken.

For transmission electron microscopy, fixed liver tissue was embedded in Spurr resin, cut in 50nm ultrathin sections, counterstained with uranyl acetate and lead citrate and examined using microscope. 10 micrographs per sample were taken to estimate % of necrotic hepatocytes and % of sinusoids presenting each of the evaluated parameters.

**RNA isolation and quantitative PCR**

RNA from cells and tissue were extracted using RNeasy mini kit (Qiagen) and Trizol (Life Technologies), respectively. RNA quantification was performed using a NanoDrop spectrophotometer. cDNA was obtained using QuantiTect reverse transcription kit (Qiagen). Real-Time PCR was performed in an ABI PRISM 7900HT Fast Real-Time PCR System, using TaqMan predesigned probes for HNF4α (Rn04339144_m1), Slcc22a1 (Rn00562250_m1), Mrp2 (Rn00563231_m1), Mrp3 (Rn01452854_m1), eNOS (Rn02132634_s1), ED1 (Rn00561129_m1), PECAM1 (Rn01467262_m1), Col1A1 (Rn01463848_m1), α-SMA (Rn01759928_g1), PDGFRβ (Rn01491838_m1), TNF-α (Rn01525859_g1), iNOS (Rn00561646_m1), IL-1 (Rn00580432_m1), IL-6 (Rn01410330_m1), Mrc1 (Rn01487342_m1), Arg1 (Rn00691090_m1), IL-10 (Rn00563409_m1), HGF (Rn00566673_m1), Wnt2 (Rn01500736_m1), Hamp (Rn00584987_m1), TIMP1 (Rn00587558_m1), TIMP2 (Rn00573232_m1), MMP2 (Rn01538170-m1), MMP9(Rn00579162_m1), PNPLA3 (Rn01502360_m1), TGF-β (Rn_01475963_m1) and GAPDH (Rn01775763_g1) as endogenous controls. Results, expressed as 2−ΔΔCt, represent the x-fold increase of gene expression compared with the young group.

**Telomere length measurement by quantitative PCR**

Telomere length was measured in the genomic DNA isolated and purified from liver tissue samples using DNeasy mini kit (Qiagen). All DNA samples were tested for purity and integrity using a Nanodrop 2000 spectrophotometer. Telomere length measurement was performed using a quantitative PCR-based method with slight modifications (Cawthon 2002). Briefly, 50 ng of DNA was mixed with either the telomere or AT-1 (single-copy gene) primer and the Power SYBR Green PCR Master Mix reagent followed by qPCR Real-Time on ABI PRISM 7900HT Fast Real-Time PCR System. The primer sequences used were: (5’→3’): T1, GGTTTTTGAGGGTGAGGGTGAGGGTGAGGGTGAGGGT T2, TCCCGACTATCCCTATCCCTATCCCTATCCCTATCCCTA; AT-1 1 ACGTGTTCTCAGCATCGACCGCTACC, AT-1 2 AGAATGATAAGGAAAGGGAACAAGAAGCCC. The relative telomere length was calculated as the ratio of telomere repeats to AT1 (a single-copy gene) (T/S ratio).

**Western Blotting**

Liver samples were processed and western blot performed as described (Guixe-Muntet et al. 2016). Used primary antibodies: P16 (554079, BD Pharmagen), SIRT1 (ab110304, Abcam), VEGFR2 (sc-315, Santa Cruz Biotech), HO-1 (ADI-SPA-896, Enzo), phosphorylated eNOS at Ser1177 (9571, Cell Signaling), total eNOS (610297, BD Transduction Laboratories), phosphorylated Moesin at Thr558 (sc-12895, Santa Cruz), total Moesin (sc-13122, Santa Cruz), stabilin-2 (MABC76, Merck Milipore), CRBP1 (sc-271208, Santa Cruz), NFκB (6956, cell signalling) and TLR4 (sc-293072, Santa Cruz) all 1:1000. Blots were revealed by chemiluminescence and protein expression was determined by densitometric analysis using the Science Lab 2001 Image Gauge (Fuji Photo Film, Düsseldorf, Germany). Blots were also assayed for GAPDH (1:5000, Sigma-Aldrich) content as standardization of sample loading.

**Histological Analysis**

Liver samples were fixed in 10% formalin, embedded in paraffin, sectioned, and slides were stained with hematoxylin and eosin (H&E) to analyze the hepatic parenchyma (Hide et al. 2016), or with Masson’s trichrome for liver fibrosis evaluation (Gracia-Sancho et al. 2011). Ten fields per slide were randomly taken and two independent researchers scored the hepatic histology using a semi-quantitative method (Jiménez-Castro et al. 2015), with minor modifications. Briefly, cytoplasmic vacuolation, nuclear pyknosis, cytoplasmic hypereosinophilia, loss of intercellular borders, necrosis and fat accumulation were scored as 0-abstent, 1-focal or 2-general, and neutrophil infiltration as 0-absent or 1-present, making a maximum final score of 13 points. Liver fibrosis was quantified as the purple-stained area per total area using the Axiovision software (De Mesquita et al. 2017).

Frozen sections were cut to 10 µm and stained with Oil Red O (Sigma Aldrich) for lipid analysis. Lipid droplets were evaluated as the red-stained area per total area using ImageJ software.

**Immunohistochemistry**

Liver samples were fixed in 10% formalin, embedded in paraffin, sectioned and processed for immunohistochemistry (IHC) or immunofluorescence (IF) as previously described (Marrone et al. 2015).

For IHC liver sections were incubated with antibodies against CD32b (sc-13271, Santa Cruz), CD31 (sc-376764, Santa Cruz), eNOS (sc-654, Santa Cruz), α-SMA (Dako), or CD163 (MCA342R, Biorad). After incubation with corresponding secondary antibodies, color development was induced by incubation with a DAB kit (Dako) and counterstained with hematoxylin. Sections were dehydrated and mounted. The specific staining was visualized and fifteen images per liver were acquired using a microscope equipped with a digital camera and the assistance of Axiovision software. The relative volume was calculated by dividing the number of points positive in sinusoidal areas by the total number of points over liver tissue (Vilaseca et al. 2017).

For IF, liver sections were incubated with antibodies against desmin (M0760, Dako), CD68 (MCA341R, Biorad), MPO (ab9535, Abcam) and H2B (ab52484, Abcam), incubated with secondary antibodies Alexa Fluor 488 or 555 (1:400, Life technologies) and 4′,6-diamino-2-fenilindol (1;3000; DAPI, Sigma-Aldrich) and mounted in Fluoromount G medium. Ten images per sample were obtained with a fluorescence microscope and percentage of positive area (Desmin) or positive cells per field (CD68, MPO/H2B) were quantified.

**Albumin and urea production**

Culture media from all experimental conditions after 24h of culture were sampled. Albumin and blood urea nitrogen (BUN) were measured using standard methods at the Hospital Clínic of Barcelona’s CORE laboratory. BUN values were converted to urea as 2.1428 mg/dl BUN =1 mg/dl urea.

**Cytochrome 4503A4 activity**

Phase I detoxification capacity of hepatocytes was analyzed using P450-Glo™ CYP4503A4 Assay following manufacturer’s instructions (V8901, Promega). Briefly, hepatocytes cultured for 24 hours were rinsed twice with DPBS and incubated with culture media containing 50 µM Luciferin-PFBE at 37°C for 4h. Then supernatant was collected and neutralized with Luciferin Detection Reagent. After incubation for 30 min at RT, plate luminescence was read in a luminometer (Orion II Microplate Luminometer, Germany). Samples luminescence was corrected subtracting background luminescence.

**Conventional bacterial study of feces and mesenteric lymph nodes (MLN)**

Samples of MLN and stool were plated on McConkey and blood agar (Materlab, Madrid, Spain) and inoculated in thioglycholate (Scharlab, Barcelona, Spain) for 24-48 hours at 37ºC. Specific microorganisms were identified by a manual biochemical test or automated system (Microscan, Baxter, Irvine, CA). GBT was defined by a positive bacteriological MLN culture (Úbeda et al. 2016).

**Endotoxemia quantification**

Endotoxemia was quantified using LAL Chromogenic Endotoxin Quantitation Kit (ThermoFisher), following manufacturer’s instructions.

**qPCR of cytokines in the ileum**

Quantification of the expression of rat genes was performed by qPCR. A snap frozen fragment of distal ileum was lysed in 1 ml of Tri-Reagent (Ambion). Total RNA was extracted and quantified. cDNA was obtained from 2 µg of total RNA from each sample (Improm-II reverse transcriptase, Promega) and 1 µl of cDNA sample was used as template for PCR with LC Fast Start DNA Master SYBRGreen I Kit (Roche Applied Science). PCR were carried out in Lightcycler 480 equipment (Roche). For each sample and experiment, triplicates were made and normalized by 28S mRNA levels. Gene expression values were calculated based on the ΔΔCt method. The results were expressed as 2-ΔΔCt referred as fold-expression compared to young rats. Primers were rat specific and designed using sequence data and Nucleotide BLAST software from the National Center for Biotechnology Information (NCBI; Bethesda, MD) database.

The primer sequences used were (5’→3’) Interferon-gamma: GGATGCTATGGAAGGAAAGAG, CAAAGAGTCTGAGGTAGAAAGAG; TNF-α: CCAGGAGAAAGTCAGCCTCCT, TCATACCAGGGCTTGAGCTCA; IL-17α: CAACCTGAAAGTCCTCAACTC; CACAGAAGGATATCTATCAGGG.

**Supplementary references**

Cawthon, R. M. (2002) Telomere measurement by quantitative PCR. *Nucleic Acids Res,* 30(10), 47e.

De Mesquita, F. C., Guixé-Muntet, S., Fernández-Iglesias, A., Maeso-Díaz, R., Vila, S., Hide, D., Ortega-Ribera, M., Rosa, J. L., Garcia-Pagán, J. C., Bosch, J., De Oliveira, J. R. & Gracia-Sancho, J. (2017) Liraglutide improves liver microvascular dysfunction in cirrhosis: Evidence from translational studies. *Sci. Rep.,* 7(1), 3255. https://doi: 10.1038/s41598-017-02866-y.

Gracia-Sancho, J., Lavina, B., Rodriguez-Vilarrupla, A., Brandes, R. P., Fernandez, M., Bosch, J. & Garcia-Pagan, J. C. (2007) Evidence against a role for NADPH oxidase modulating hepatic vascular tone in cirrhosis. *Gastroenterology*, 133(3), 959-66. https://doi: 10.1053/j.gastro.2007.06.021.

Gracia-Sancho, J., Russo, L., García-Calderó, H., García-Pagán, J. C. , García-Cardeña, G. & Bosch, J. (2011) Endothelial expression of transcription factor Kruppel-like factor 2 and its vasoprotective target genes in the normal and cirrhotic rat liver. *Gut*, 60(4), 517-24. https://doi: 10.1136/gut.2010.220913.

Guixe-Muntet, S., de Mesquita, F. C., Vila, S., Hernandez-Gea, V., Peralta, C., Garcia-Pagan, J. C., Bosch, J. & Gracia-Sancho, J. (2016) Cross-talk between autophagy and KLF2 determines endothelial cell phenotype and microvascular function in acute liver injury. *J. Hepatol,* 66(1), 86-94. https://doi: 10.1016/j.jhep.2016.07.051.

Hide, D., Ortega-Ribera, M., Garcia-Pagan, J. C., Peralta, C., Bosch, J. & Gracia-Sancho, J. (2016) Effects of warm ischemia and reperfusion on the liver microcirculatory phenotype of rats: Underlying mechanisms and pharmacological therapy. *Sci. Rep.,* 6, 22107. https://doi: 10.1038/srep22107.

Jiménez-Castro, M. B., Meroño, N., Mendes-Braz, M., Gracia-Sancho, J., Martínez-Carreres, L., Cornide-Petronio, M. E., Casillas-Ramirez, A., Rodés, J. & Peralta, C. (2015) The effect of brain death in rat steatotic and non-steatotic liver transplantation with previous ischemic preconditioning. *J. Hepatol.,* 62, 83–91. https://doi: 10.1016/j.jhep.2014.07.031.

Le Couteur, D. G., Cogger, V. C., Markus, A. M. A., Harvey, P. J., Yin, Z. L., Ansselin, A. D. & McLean, A. J. (2001) Pseudocapillarization and associated energy limitation in the aged rat liver. *Hepatology*, 33(3), 537-43. https://doi: 10.1053/jhep.2001.22754.

Marrone, G., Maeso-Díaz, R., García-Cardena, G., Abraldes, J. G., García-Pagán, J. C., Bosch, J. & Gracia-Sancho, J. (2015) KLF2 exerts antifibrotic and vasoprotective effects in cirrhotic rat livers: Behind the molecular mechanisms of statins. *Gut*, 64(9),1434-43. https://doi: 10.1136/gutjnl-2014-308338.

Úbeda, M., Lario, M., Muñoz, L., Borrero, M. J., Rodríguez-Serrano, M., Sánchez-Díaz, A. M., … Albillos, A. (2016) Obeticholic acid reduces bacterial translocation and inhibits intestinal inflammation in cirrhotic rats. *J. Hepatol.,* 64, 1049–1057. https://doi: 10.1016/j.jhep.2015.12.010.

Vilaseca, M., García-Calderó, H., Lafoz, E., Ruart, M., López-Sanjurjo, C., Murphy, M. P., … García-Pagán, J. C. (2017) Mitochondria-targeted antioxidant mitoquinone deactivates human and rat hepatic stellate cells and reduces portal hypertension in cirrhotic rats. *Liver Int.,* 37, 1002–1012. https://doi: 10.1111/liv.13436.

**Supplementary table 1**

Severity of hepatic injury features in 3 months-young and 20 months-old rats.

| **Parameter** | **3 months-Young** | **20 months-Old** | **p-value** |
| --- | --- | --- | --- |
| Cytoplasmic vacuolation | 0.46 ± 0.15 | 1.33 ± 0.14 | **< 0.001** |
| Nuclear pyknosis | 0.31 ± 0.14 | 0.89 ± 0.08 | **< 0.001** |
| Cytoplasmic hypereosinophilia | 0.31 ± 0.14 | 0.89 ± 0.11 | **< 0.001** |
| Loss of intercellular borders | 0.08 ± 0.08 | 0.78 ± 0.13 | 0.07 |
| Necrosis | 0.15 ± 0.11 | 0.44 ± 0.12 | **0.02** |
| Neutrophil infiltration | 0.31 ± 0.14 | 0.72 ± 0.11 | **< 0.001** |
| Fat accumulation | 0.64 ± 0.22 | 1.33 ± 0.14 | **< 0.001** |
| **Final Score** | **2.15 ± 0.64** | **6.39 ± 0.43** | **< 0.001** |

Data expressed as mean ± SEM (n = 12 each group).

**Supplementary table 2**

Clinical characteristics of human liver donors.

| **Parameter** | **Young (n=14)** | **Old (n=13)** | **p-value** |
| --- | --- | --- | --- |
| Age (years) | 28.86 ± 2.00 | 76.14 ± 0.87 | **<0.001** |
| Body mass index (kg/cm^2^) | 26.26 ± 1.48 | 26.19 ± 0.67 | >0.2 |
| Hepatic steatosis (%) | 4.71 ± 2.33 | 4.24 ± 2.12 | >0.2 |
| ICU stay (h) | 66.00 ± 23.07 | 61.60 ± 12.07 | >0.2 |
| Gender (% female) | 14 | 30 | >0.2 |
| Cause of death (%)  Hemorrhagic stroke  Ischemic stroke  Traumatic Brain Injury  Hypoxia  Others | 31  15  23  23  8 | 92  0  8  0  0 |  |

Data expressed as mean ± SEM.

**Supplementary Figure Legends**

**Supplementary Fig.1. Senescence markers in 3 months-young and 20 months-old rats.** P16 protein expression in liver tissue (A), primary LSEC (B) and primary HSC (C), normalized to corresponding GAPDH. (D) SIRT1 protein expression in liver tissue normalized to GAPDH. (E) Telomere length from young and old liver tissue measured as the ratio of telomere repeats to a single-copy gene. n=12 (A and D) and n=6 (B, C and E) per group. Results represent mean ± S.E.M.

**Supplementary Fig.2. Hepatic architecture in 3 months-young and 20 months-old rats.** Representative images of hematoxylin & eosin staining in liver tissue (100X). n=12 per group.

**Supplementary Fig.3. Liver injury and hepatocyte function in 3 months-young and 20 months-old rats.** (A) TUNEL staining and quantification from young and old livers (200X). (B) Urea and albumin production from freshly isolated young and old hepatocytes. (C) HNF4α, Slc22a1, Mrp2 and Mrp3 mRNA expression in livers described in B. (D) Cytochrome P4503A4 activity from cells described in B. n=12 (A and C) and n=6 (B and D) per group. Results represent mean ± S.E.M.

**Supplementary Fig.4. HSC evaluation in healthy young and old human livers.** Representative images of desmin immunofluorescence (A) and α-SMA immunohistochemistry (B) with their corresponding quantification from young and old human livers. n=14 young & 13 old livers. Results represent mean ± S.E.M. All images 400X, scale bar=50μm.
